# Structure–Function Interplay in Piezoelectric PCL/BaTiO_3_ Scaffolds Fabricated by Phase Separation: Correlation of Morphology, Mechanics, and Cytocompatibility

**DOI:** 10.3390/ijms27010406

**Published:** 2025-12-30

**Authors:** Abdulkareem Alotaibi, Yash Desai, Jacob Miszuk, Jae Hyouk Choi, Konstantinos Michalakis, Alexandros Tsouknidas

**Affiliations:** Department of Restorative Sciences & Biomaterials, Henry M. Goldman School of Dental Medicine, Boston University, Boston, MA 02118, USA; keem94@bu.edu (A.A.); dryash@bu.edu (Y.D.); jmmiszuk@bu.edu (J.M.); jaehchoi@bu.edu (J.H.C.); kmich@bu.edu (K.M.)

**Keywords:** piezoelectric composites, phase separation fabrication, cell adhesion and proliferation, biomechanical performance, biological performance

## Abstract

Bone regeneration relies on the coordinated interplay between mechanical and biological cues. Piezoelectric composites, capable of converting mechanical strain into electrical signals, offer a promising approach to stimulate osteogenesis. This study aimed to develop and characterize polycaprolactone (PCL) and barium titanate (BaTiO_3_) composite scaffolds fabricated through thermally induced phase separation (TIPS), and to systematically evaluate the effects of polymer concentration and ceramic incorporation on scaffold morphology, porosity, mechanical properties, and cytocompatibility were systematically evaluated. The resulting scaffolds exhibited a highly porous, interconnected architecture, with 9% PCL formulation showing the most uniform morphology and consistent mechanical and biological behavior. Incorporation of BaTiO_3_ did not alter pore structure or compromise cytocompatibility but slightly enhanced stiffness and surface uniformity. SEM-based image analysis confirmed homogeneous BaTiO_3_ dispersion across all formulations. MTT assays and confocal microscopy demonstrated robust pre-osteoblast adhesion and spreading, particularly on denser composite scaffolds, confirming that the inclusion of BaTiO_3_ supports a favorable environment for cell proliferation. Overall, optimizing polymer concentration and ceramic dispersion enables fabrication of structurally coherent, cytocompatible scaffolds. The findings establish structure–property–biology relationships that serve as a baseline for future investigations into the electromechanical behavior of PCL/BaTiO_3_ scaffolds and their potential to promote osteogenic differentiation under physiological loading.

## 1. Introduction

Alveolar bone, a specialized tooth-dependent tissue, forms in conjunction with tooth development, followed by continuous remodeling in response to tooth eruption and functional stimuli transduced through the dentition [1]. A major challenge in oral and craniofacial health is the progressive resorption and atrophy of alveolar bone following tooth loss or extraction, which leads to marked reductions in bone volume and quality [2]. The effect of this resorption is most notable in the anterior maxilla, where continued resorption leads to significant thinning of the labial plate [3]. The resulting reduction in ridge height and width often compromises bone availability for implant placement and aesthetic restoration [1,2]. In such cases, achieving adequate primary stability and ideal prosthetic positioning becomes challenging, frequently necessitating bone augmentation or the use of shorter and angulated implants [4].

Tissue engineering has become a promising interdisciplinary approach to restore damaged tissues through the integration of cell-seeded scaffolds and/or controlled delivery of signaling molecules [5]. Among the diverse applications of tissue engineering, bone regeneration has gained particular attention due to the prevalence of osseous defects resulting from trauma, disease, and surgical intervention [5]. Traditionally, bone regeneration has relied on autografts, which face inherent limitations such as donor site morbidity [6]. Grafting techniques, employing allografts and xenografts instead, are subject to immunogenicity and incomplete or delayed integration, largely due to limited recruitment of osteogenic cells and insufficient biological signaling [7]. Recognizing that bone regeneration is inherently a mechanobiological process, recent strategies emphasize scaffolds engineered to modulate the cellular microenvironment and enhance endogenous regeneration through biomechanical and electromechanical cues [8].

Polycaprolactone (PCL) has emerged as a widely utilized polymer for the fabrication of scaffolds that reproduce the structure and function of natural bone matrices. As a material, PCL offers biocompatibility, biodegradability, and favorable mechanical properties, while it can be processed through a variety of fabrication techniques such as phase separation and additive manufacturing, i.e., electrospinning and Fused Deposition Modeling (FDM) [9]. Its slow degradation rate and structural stability make it particularly suitable for dental and craniofacial applications where long-term mechanical support is essential [10].

Beyond its intrinsic material advantages, the design and fabrication parameters of PCL strongly determine the resulting scaffold architecture and its biological performance. The physical properties of a scaffold (particularly its pore size, shape, and morphology) play a critical role in modulating cell adhesion, nutrient diffusion, and the mechanotransduction of forces to resident cells, thereby influencing both its mechanical stability (i.e., its equivalent bulk Young’s modulus) and biological performance [11]. Building on these structure–function relationships, the incorporation of piezoelectric ceramics, or polymers, enables the design of scaffolds that convert mechanical stimuli into localized electrical signals, actively promoting osteogenic differentiation and tissue regeneration.

Bone is intrinsically piezoelectric, generating localized electrical potentials under mechanical loading that regulate remodeling and repair. Replicating this electromechanical environment has therefore become a central objective in bone tissue engineering. By integrating piezoelectric materials into scaffold design, it is possible to replicate electrical signaling pathways that naturally guide osteogenesis [12]. Among these materials, ceramics such as barium titanate (BaTiO3) and conductive polymers like poly(3,4-ethylenedioxythiophene) have shown promise in supporting mineralization and matrix organization within engineered constructs, without requiring external biochemical factors [13].

In recent years, PCL/BaTiO_3_ composite systems have been increasingly explored as candidate piezoelectric scaffolds for bone tissue engineering, leveraging the favorable processability and biocompatibility of PCL together with the piezoelectric properties of BaTiO_3_. Previous work has demonstrated the feasibility of fabricating PCL/BaTiO_3_ scaffolds with measurable piezoelectric response and promising osteogenic activity in vitro [14,15] and, more broadly, piezoelectric biomaterials have been reviewed as smart scaffolds that can provide electrical cues to cells in regenerative contexts.

The present study aims to develop and characterize piezoelectric composite scaffolds based on polycaprolactone (PCL) and barium titanate (BaTiO_3_) fabricated via a rapid and cost-effective phase-separation technique. The investigation is designed as a focused case study on thermally induced phase separation (TIPS) process optimization, examining how polymer concentration and ceramic incorporation define a mechanically and biologically viable processing window for porous PCL/BaTiO_3_ scaffolds.

Our null hypothesis (H_0_) is that the structural and mechanical properties of PCL/BaTiO_3_ scaffolds do not significantly affect cell adhesion and viability, while our alternative hypothesis (H_1_) is that optimization of the structural and mechanical properties of PCL/BaTiO_3_ scaffolds enhances cell adhesion and viability, thereby providing a foundation for future studies on their electromechanical performance under dynamic loading.

## 2. Results

**Figure 1:**
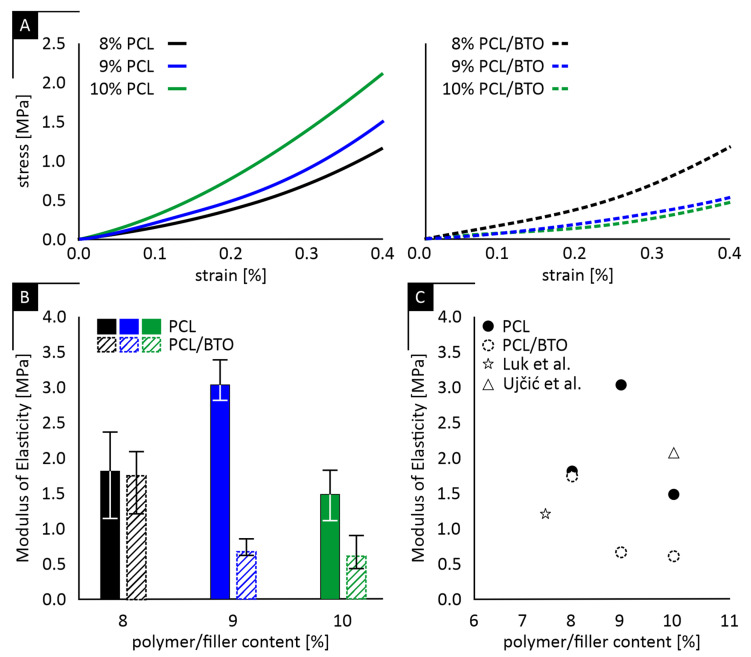
Mechanical characterization of scaffolds. (**A**) Representative compressive stress–strain curves for PCL and PCL/ BaTiO_3_ scaffolds. (**B**) Elastic modulus as tested across all compositions. Incorporation of BaTiO_3_ slightly modified stiffness across polymer concentrations, without a consistent trend. (**C**) Comparison of the calculated moduli to literature data with comparable compositions [16,17].

Representative compressive stress–strain curves for PCL and PCL/BaTiO_3_ scaffolds are shown in Figure 1A, while the corresponding elastic moduli are summarized in Figure 1B. It should be noted that scaffolds fabricated at polymer concentrations of 5–7% PCL exhibited insufficient macroscopic structural integrity during handling and demolding, including partial collapse and poor load-bearing stability. These limitations precluded reliable mechanical and biological testing; therefore, subsequent characterization was focused on the 8–10% PCL formulations, which exhibited a nonlinear stress–strain response typical of highly porous polymeric materials.

The mean (μ) elastic modulus of PCL scaffolds was 1.82 with a standard deviation (σ) of ± 0.63 MPa, 3.06 ± 0.30 MPa, and 1.49 ± 0.37 MPa for 8%, 9%, and 10% polymer concentrations, respectively. The addition of BaTiO_3_ produced corresponding mean values of 1.73 ± 0.46 MPa, 0.68 ± 0.13 MPa, and 0.59 ± 0.25 MPa for the same polymer concentrations. Reproducibility, assessed through the coefficient of variation (CV% = (σ/μ) × 100), ranged from ≈ 10% to >40% among groups, indicating variable consistency in scaffold fabrication and mechanical performance. The 9% PCL group showed the lowest variability (≈10%), whereas higher dispersion was observed in the 8% and 10% formulations, particularly in the 10% PCL + BaTiO_3_ condition (CV > 40%). This trend is consistent with concentration-dependent phase-separation behavior in TIPS systems, where lower polymer contents may lead to insufficient chain entanglement and mechanically weaker struts, while higher concentrations increase solution viscosity and promote earlier gelation, restricting phase separation and increasing architectural heterogeneity. Statistical comparison of the mean moduli using one-way ANOVA followed by Tukey’s post hoc test revealed significant differences (*p* < 0.05) between several groups, confirming that variations in mechanical stiffness are not attributable to measurement noise but reflect intrinsic differences in scaffold architecture and composition. Figure 1C further summarizes the relationship between porosity and elastic modulus across all scaffold formulations and scaffolds of comparable compositions found in literature. The plot highlights the inverse trend between structural density and stiffness, where denser (less porous) scaffolds exhibit higher modulus values. This relationship provides a quantitative overview linking fabrication parameters to resulting mechanical performance.

Analytical predictions derived from the Gibson–Ashby porosity–modulus relation systematically underestimated the experimentally measured elasticity for all groups, as summarized in Table 1 [18]. This pronounced deviation confirms that porosity alone cannot account for the mechanical performance of the fabricated scaffolds. The limited correlation between analytical predictions and experimental stiffness values indicates that factors beyond the nominal void fraction—such as pore morphology, inter-pore connectivity, and local densification of struts—govern the effective mechanical response.

**Figure 2:**
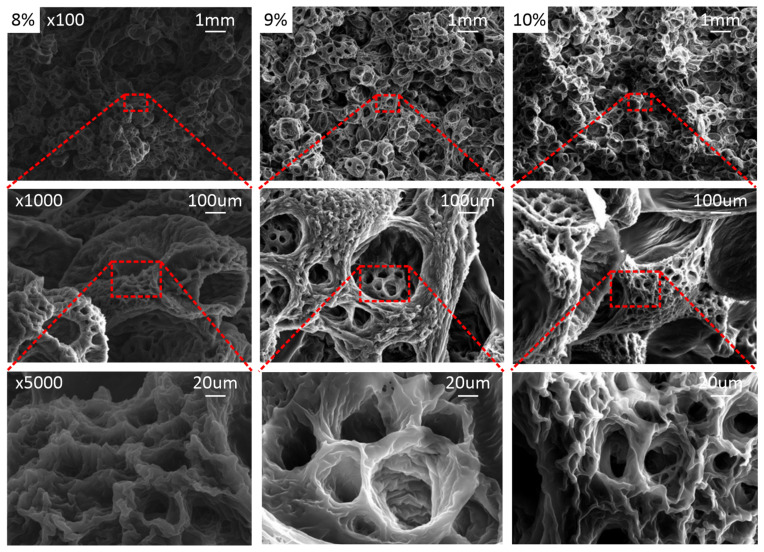
Representative SEM morphology of PCL scaffolds fabricated by TIPS at 8%, 9%, and 10% polymer-to-solvent concentrations. Consecutive magnifications are presented for each sample, with scale bars of 1 mm (100×), 100 μm (1000×), and 20 μm (5000×). Increasing polymer concentration resulted in denser and more continuous structures with reduced pore size and enhanced surface definition.

**Figure 3:**
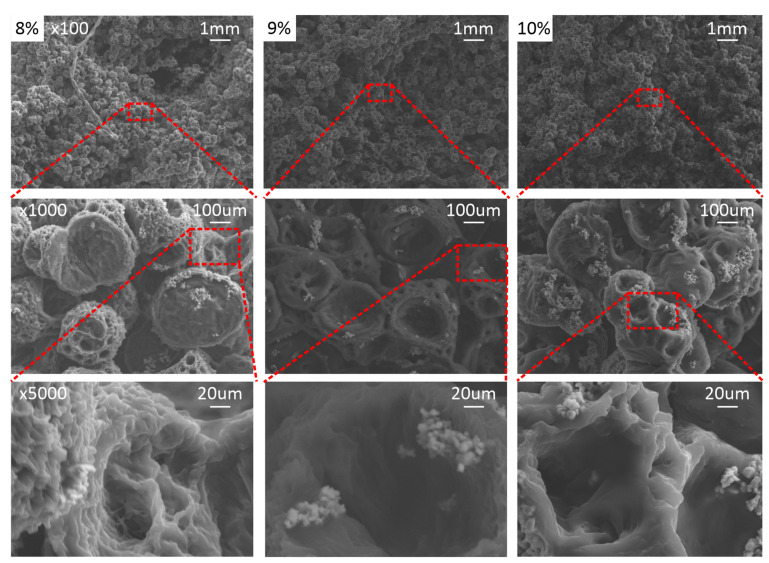
Representative SEM morphology of PCL/BaTiO_3_ composite scaffolds fabricated via TIPS at 8%, 9%, and 10% combined polymer + filler to solvent concentrations. Consecutive magnifications are presented for each sample, with scale bars of 1 mm (100×), 100 μm (1000×), and 20 μm (5000×). The incorporation of BaTiO_3_ nanoparticles preserved the overall porous architecture while promoting smoother internal surfaces and localized particle clusters within pore walls.

To substantiate these interpretations, scanning electron microscopy (SEM) was conducted to examine the underlying architecture of mechanically viable scaffolds, with and without BaTiO_3_, as illustrated in Figure 2 and Figure 3 for PCL-only and PCL/BaTiO_3_ scaffolds, respectively. Notably, these analyses focused only on the 8–10% PCL range, which exhibited sufficient mechanical coherence for further characterization. Representative SEM micrographs of scaffolds fabricated at lower polymer concentrations (5–7% PCL) are provided for contextual completeness in the Supplementary Material.

In line with the data presented in Table 1, increasing polymer concentration produced denser, more continuous structures with reduced pore size and enhanced surface definition. The 9% formulations exhibited the most uniform and well-defined pore morphology, while the 8% and 10% groups displayed less cohesive networks.

These observations suggest that the load-bearing walls behave as locally densified, composite-like domains rather than ideal open-cell struts. Variations in strut thickness, pore shape anisotropy, and spatial distribution within the scaffold architecture likely enhance load-transfer efficiency, thereby explaining the consistently higher experimental moduli compared to analytical predictions. Indicative pore size estimation from repeated SEM measurements of the selected polymer-to-solvent formulation yielded a mean pore size of approximately 35 µm, with a distribution ranging from 10 to 70 µm.

**Figure 4:**
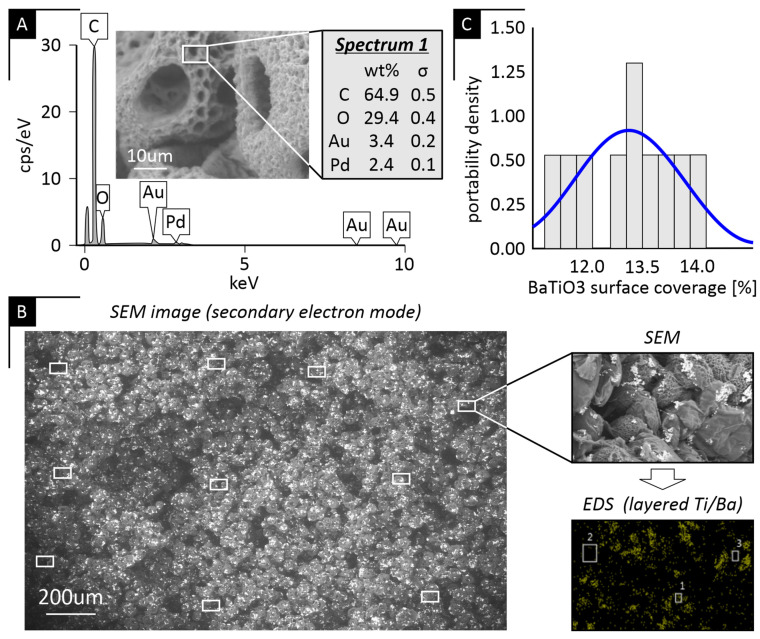
Quantitative analysis of BaTiO_3_ dispersion: (**A**) Representative EDS spectrum confirming the elemental composition of the PCL/BaTiO_3_ scaffold. (**B**) SEM image (secondary electron mode) showing the measurement locations used for BaTiO_3_ surface quantification across a scaffold cross-section, together with a representative backscattered electron (BSE) image highlighting uniform BaTiO_3_ dispersion. (**C**) Indicative histogram (and trendline) of surface coverage values derived from ImageJ 1.54r threshold analysis, demonstrating homogeneous BaTiO_3_ distribution across analyzed regions.

A representative EDS spectrum of the PCL-only scaffold (Figure 4A) confirmed the absence of Ba and Ti peaks in the polymer baseline, while the corresponding SEM image (Figure 4B) illustrates the measurement locations used for quantitative surface analysis in the 9% PCL/BaTiO_3_ scaffold. The accompanying backscattered electron (BSE) micrograph shows an even distribution of BaTiO_3_ particles without detectable agglomeration or localized enrichment. Quantitative evaluation of ten surface regions of interest (ROIs) per composite scaffold revealed a narrow, near-normal distribution of BaTiO_3_ surface coverage with minimal regional variability (Figure 4C). Across all composites, mean BaTiO_3_ coverage values were consistent among the 8%, 9%, and 10% groups, and statistical analysis (*p* > 0.05) confirmed that polymer concentration did not affect filler dispersion (Table 2). Collectively, these findings demonstrate uniform BaTiO_3_ incorporation and confirm the reproducibility of the fabrication process.

It should be noted that all SEM, BSE, and EDS analyses were conducted on fractured scaffold cross-sections; therefore, the reported BaTiO_3_ surface coverage reflects filler distribution within the internal pore walls throughout the scaffold volume, rather than being limited to the external scaffold surfaces, while EDS was used to qualitatively confirm BaTiO_3_ presence at selected locations.

Cell viability on the fabricated scaffolds was evaluated using metabolic MTT assays. For comparative consistency, cell-viability data are presented as absorbance values (OD_590_) rather than percentage cytotoxicity (see Table 3), since all scaffolds exhibited values within the non-toxic range (cell viability > 85%).

All scaffold types exhibited comparable metabolic activity (*p* > 0.05), confirming good cytocompatibility for both PCL and PCL/BaTiO_3_ composites. A modest increase in metabolic activity was observed for the 9% PCL/BaTiO_3_ group relative to the other formulations; however, this is reported merely as an observation and not interpreted as evidence of a specific underlying biological mechanism. Overall, these findings demonstrate that BaTiO_3_ incorporation did not compromise cell viability, and that all scaffold formulations provided a favorable surface for pre-osteoblast attachment and growth.

**Figure 5:**
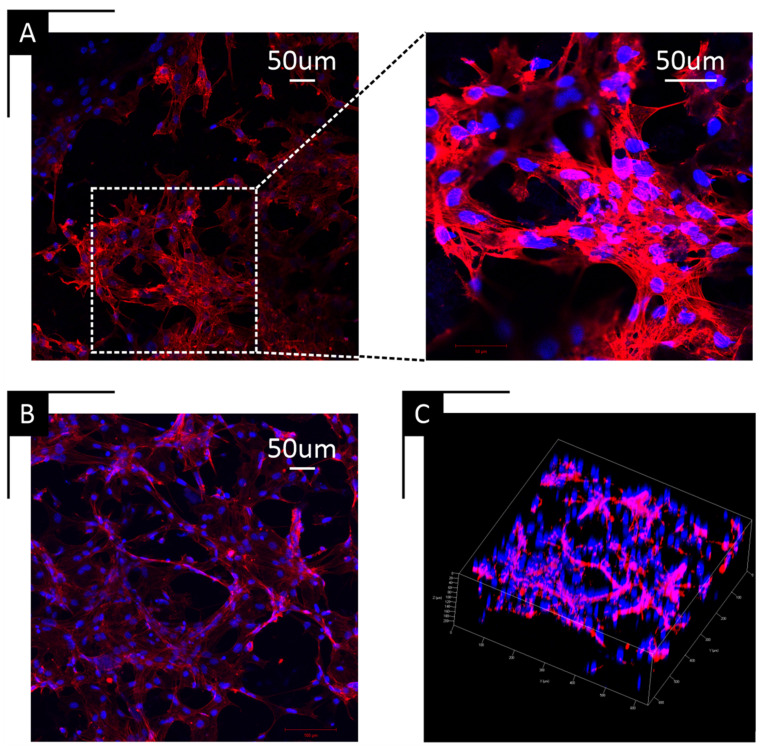
Confocal microscopy of cell–scaffold interactions. High-magnification confocal micrographs showing cytoskeletal organization and cell spreading on scaffold surfaces. Representative images are shown for (**A**) 10% PCL/BaTiO_3_ composite scaffold, (**B**) 10% PCL-only scaffold, and (**C**) three-dimensional reconstruction of the 10% PCL/BaTiO_3_ composite scaffold, illustrating cell infiltration within the pore network.

To complement the MTT data, confocal microscopy confirmed cell attachment and cytoskeletal organization across all scaffold types, as shown indicatively for the 10% PCL/BaTiO_3_ (Figure 5A) and 10% PCL-only scaffolds (Figure 5B). Actin staining (red) showed well-defined cytoskeletal networks and widespread cell coverage, with nuclei counterstained in blue. Three-dimensional reconstruction images (Figure 5C) showed cellular infiltration throughout the scaffold depth, confirming that the porous structure supported cell migration and colonization beyond the surface layer.

## 3. Discussion

The present study investigated how polymer (PCL) concentration and the incorporation of a functional piezoelectric ceramic (BaTiO_3_) influence the morphology, mechanical response, and biological performance of scaffolds fabricated by thermally induced phase separation (TIPS). Because these composites are ultimately intended for electromechanically active bone tissue engineering applications, understanding how processing parameters affect scaffold architecture and stiffness is essential; both properties directly govern the distribution of mechanical strain and, consequently, the magnitude of piezoelectric cues generated under dynamic loading.

The cumulative results highlight that even subtle variations in fabrication conditions can meaningfully alter scaffold integrity and performance—a sensitivity that is well documented in phase-separated and solvent-cast polymer systems, where small changes in polymer concentration, solvent ratio, or cooling rate significantly impact pore morphology, interconnectivity, and downstream mechanical behavior [19]. Nevertheless, within the range of fabrication parameters examined, the scaffolds consistently demonstrated reproducible morphology, stable mechanical behavior, and robust cytocompatibility, supporting their suitability for bone tissue engineering applications.

Mechanical testing revealed non-linear stress–strain behavior typical of porous polymers, with stiffness values that did not corelate directly with polymer concentration or BaTiO_3_ inclusion, a trend consistent with previous reports on TIPS-derived scaffolds [20]. Although increasing polymer content reduced overall porosity, the elastic modulus fluctuated rather than increasing monotonically. We hypothesize that higher polymer concentrations increase solution viscosity and promote earlier gelation, thereby altering phase-separation kinetics and leading to greater architectural heterogeneity. This interpretation aligns with observations in PLLA–dioxane–water TIPS systems, where higher polymer concentrations lead to earlier gelation and crystallization that restrict the liquid–liquid phase-separation process, resulting in denser structures with smaller and less interconnected pores [21]. Conversely, at lower polymer concentrations, the reduced chain entanglement likely produced thinner and less continuous strut walls, weakening the load-bearing framework despite the higher void fraction. This rationale is consistent with well-established polymer-solution behavior, where insufficient molecular entanglement at low concentrations leads to discontinuous or unstable structural domains, as reported by Shenoy et al. [22] for dilute polymer systems.

These findings suggest that the mechanical performance of TIPS-derived scaffolds is governed not by bulk density alone, but by the continuity and uniformity of the pore walls. The analytical comparison using the Gibson–Ashby relation further supports this interpretation: the experimental moduli were consistently higher than theoretical predictions, indicating that the load-bearing struts behaved as locally densified, composite-like domains rather than as ideal open-cell foams. Within this context, the 9% PCL scaffolds demonstrated the most consistent stiffness values, implying that this polymer concentration provides a balance between adequate chain entanglement and efficient phase-separation kinetics, enabling the formation of more coherent and mechanically effective pore architecture. This observation aligns with what Onder et al. reported in 2018, showing that intermediate PCL concentration (8–10 wt%) provides sufficient chain entanglement without hindering phase separation, yielding more uniform pore structures and stable mechanical properties [23].

The SEM findings provide microstructural support for these mechanical interpretations. At 8% polymer concentration, the scaffolds exhibited fragmented, cotton-ball-like pore assemblies, consistent with insufficient chain entanglement and an unstable phase-separation front—features that explain the reduced and inconsistent stiffness observed experimentally. In contrast, the 10% scaffolds displayed denser yet more irregular pore walls, in line with the notion that earlier gelation at higher polymer content restricts liquid–liquid demixing and prevents the development of well-connected pore channels. Comparable concentration-dependent trends have been reported by Aydin et al. [24], reinforcing that polymer wt% critically governs TIPS morphology. Notably, the 9% PCL and PCL/BaTiO_3_ scaffolds showed the most uniform and continuous pore architecture, directly supporting the mechanical finding that this intermediate concentration achieves a more favorable balance between polymer mobility and phase-separation kinetics.

The uniform distribution of BaTiO_3_ throughout the PCL matrix, as verified by SEM, BSE imaging, and quantitative surface analysis, further supports that the ceramic phase did not interfere with phase separation or alter pore continuity. Airimioaei et al. [25] reported similar findings in their paper, as they demonstrated that PCL/BaTiO_3_ composites containing 10 vol% BaTiO_3_ exhibit a homogenous and uniform microstructure and good BaTiO_3_ dispersion. The absence of agglomeration and the near-normal surface distribution curve confirmed homogeneous incorporation of the ceramic filler, underscoring the reproducibility of the composite fabrication process. The consistent filler dispersion across concentrations suggests that variations in scaffold stiffness are primarily attributable to polymer-phase morphology rather than particle distribution.

Biological characterization showed comparable metabolic activity across all scaffold groups, indicating that BaTiO_3_ incorporation preserved cytocompatibility while potentially enhancing surface interactions, in agreement with Liu et al. [26]. The modest increase in cell proliferation observed for the 9% PCL/BaTiO_3_ group is consistent with its more favorable structural and mechanical features, reinforcing the link between scaffold architecture and cellular response. Confocal microscopy further supported this relationship by demonstrating extensive cytoskeletal spreading on composite scaffolds, with well-organized actin filaments spanning pore walls and penetrating the three-dimensional network—evidence of effective surface adhesion and sufficient pore interconnectivity to support cell infiltration.

From a translational perspective, the present findings align with key requirements for clinical adoption of PCL-based scaffolds in craniofacial and dental applications. Previous clinical and preclinical studies, as summarized in recent translational reviews of PCL-based scaffolds for craniofacial applications [10], indicate that PCL scaffolds can provide sufficient mechanical stability for defect support, exhibit favorable handling characteristics, and be manufactured in a reproducible and scalable manner for patient-specific applications. Within this context, the present study demonstrates that TIPS-fabricated PCL/BaTiO_3_ scaffolds can achieve mechanically stable, highly porous architectures while preserving cytocompatibility, thereby addressing critical design criteria for guided bone regeneration and alveolar ridge augmentation. It should be noted that while TIPS is frequently described in the literature as amenable to scale-up due to its simplicity and low processing requirements, the present work does not experimentally evaluate manufacturing scalability. Accordingly, scalability is discussed here in a qualitative and prospective manner, consistent with the scope of this study as a focused case study on TIPS process optimization rather than a comprehensive manufacturing assessment.

As highlighted in recent translational reviews, the clinical impact of polymeric scaffolds depends on achieving an appropriate balance between mechanical integrity, degradation kinetics, and biological performance, rather than maximizing any single material property [20]. The structure–property relationships identified here therefore provide practical design guidance for next-generation electromechanically active scaffolds intended to augment existing clinical workflows rather than replace them.

While the present study establishes clear structure–property relationships, the analytical treatment of scaffold mechanics was intentionally limited to porosity-based models. Future work could extend predictive frameworks by incorporating morphology descriptors derived either from image-based analyses (micro-CT) or advanced pore size and swelling-dependent analyses [27] to include strut thickness distributions, pore connectivity, and architectural anisotropy. From a biological perspective, future studies will also focus on longer-term cell culture and differentiation assays to assess whether the modest differences in short-term metabolic activity observed here persist over time or translate into functional osteogenic outcomes. Targeted experiments aimed at decoupling the effects of scaffold architecture, local stiffness, and surface chemistry will be required to clarify their respective contributions to cell behavior. In addition, future work will include direct electromechanical and piezoelectric characterization (e.g., d_33_ measurements and electromechanical coupling under dynamic loading), building upon the structural and biological baseline established in the present study, as well as a systematic investigation of BaTiO_3_ concentration effects to better elucidate the coupled mechanical and electromechanical behavior of the composite scaffolds.

## 4. Materials and Methods

### 4.1. Scaffold Fabrication

Porous polycaprolactone (PCL) and PCL/barium titanate (BaTiO_3_) composite scaffolds were fabricated using a thermally induced phase separation (TIPS) method, following procedures adapted from Yao et al. [14]. Briefly, PCL (Mw = 80,000; Polysciences, Warrington, PA, USA) was dissolved in a binary solvent system consisting of tetrahydrofuran (THF) (≥99.0%, containing 250 ppm BHT; Sigma-Aldrich, St. Louis, MO, USA) and deionized water at polymer-to-solvent concentrations ranging from 5–10% (*w*/*w*), increased in 1% increments under continuous stirring at 50 °C for 24 h to ensure complete polymer dissolution. For composite groups, a single BaTiO_3_ loading of 10 wt% BaTiO_3_ microparticles (<3 μm, 99%; Sigma-Aldrich) relative to the polymer mass was added and homogenized using a magnetic stirrer until uniform dispersion was achieved. The resulting solutions were cast into cylindrical molds and cooled at −20 °C to induce phase separation. Samples were subsequently lyophilized for 24 h and stored in a desiccator prior to characterization.

### 4.2. Mechanical Evaluation (Compression Test)

Compressive testing was performed on an Instron 5566A universal testing machine (Instron, Norwood, MA, USA) equipped with a 100 N load cell. Cylindrical specimens (Ø = 5 mm, h = 3 mm) were compressed at 1 mm/min, up to 50% strain. Engineering stress–strain curves were obtained by normalizing load and displacement to the initial cross-sectional area and height, respectively. Because of the stochastic scaffold architecture and their anisotropy, an effective longitudinal elastic modulus (*E*) was calculated from the initial linear region of the stress–strain curve (small-strain tangent of Figure 1). Each condition was tested in triplicate, and the reported modulus per group represents the mean ± standard deviation of three specimens.

To contextualize the experimental moduli and assess fabrication quality and structure–property alignment, the results were compared with an established high-porosity (foam) relation [15] using the equivalent pore volume fraction f (total void fraction of the specimen), as shown in Equation (1):

E_porous_ = E_bulk_ (1 − *f*)^2^ (1)

where E_“bulk” is the elastic modulus of the solid wall material. For all groups (PCL and PCL/BaTiO_3_), E_“bulk” was set to 1.36 MPa (bulk PCL). No correction was applied for the inclusion of BaTiO_3_ microparticles, as their low volume fraction (≈2 vol %) was assumed to produce a negligible effect on the wall modulus at the strut scale.

### 4.3. Density and Porosity Calculation

The bulk density ($p_{\mathrm{bulk}}$) of PCL and BaTiO_3_ was determined using a gas pycnometer (AccuPyc II 1340; Micromeritics, Norcross, GA, USA) at room temperature. The dry mass (*m*) of each scaffold was measured using an analytical balance with 0.0001 g accuracy (Mettler Toledo, Greifensee, Switzerland), while the external geometric volume (*V*) was determined using a high-resolution dental laboratory scanner (inEos X5; Dentsply Sirona, Charlotte, NC, USA). Based on these measurements, scaffold porosity (*P*) was calculated according to Equation (2):

(2) $$P=1-\frac{m}{p_{\mathrm{bulk}}V}$$

This method provided accurate estimation of total void fraction by comparing the apparent scaffold density with the theoretical bulk density of the polymer matrix. Porosity data were used to interpret structure–property relationships, particularly the correlation between scaffold density and compressive modulus.

### 4.4. Scanning Electron Microscopy (SEM)

The morphology and pore structure of the scaffolds were examined using a field-emission scanning electron microscope (FE-SEM; SU6600, Hitachi, Tokyo, Japan). Samples were frozen at −80 °C, fractured to expose the internal cross-section, and sputter-coated with a gold/palladium alloy (75/25) using a Technics Hummer V coater (Anatech Ltd., Clearwater, FL, USA). Imaging was performed under high vacuum at 15 kV in secondary electron (SE) mode to evaluate surface topography and backscattered electron mode to assess BaTiO_3_ particle distribution and contrast within the polymer matrix. Representative micrographs were captured at 100×, 1000×, and 5000× magnification. Pore size and BaTiO_3_ dispersion were assessed using ImageJ software version 1.54r (NIH, Bethesda, MD, USA).

### 4.5. Scaffold Preparation and Sterilization for Cell Culture

Prior to cell culture, a biopsy punch was used to prepare scaffold samples (Ø = 3 mm, h = 2 mm), sterilized in 70% ethanol for 30 min, and rinsed three times at 10 min each with phosphate-buffered saline (PBS, pH 7.4). Scaffolds were pre-incubated in Minimum Essential Medium (MEM; Gibco, Waltham, MA, USA) for 15 min to enhance protein adsorption and promote cell attachment.

### 4.6. MTT Assay

Cell viability was assessed using the MTT Cell Proliferation Assay Kit (ab211091; Abcam, Cambridge, UK) following the manufacturer’s protocol. MC3T3-E1 pre-osteoblasts were seeded in 24-well plates (10,000 cells/well) and cultured in growth medium supplemented with 10% fetal bovine serum and 1% penicillin-streptomycin (10,000 Units/mL Penicillin + 10,000 µg/mL Streptomycin; Gibco, Thermo Fisher Scientific, Waltham, MA, USA) for 24 h at 37 °C under 5% CO_2_ to allow attachment. Scaffolds were then placed in each well and incubated for an additional 36 h. The culture medium was replaced with 50 µL serum-free medium and 50 µL MTT reagent, followed by incubation for 3 h at 37 °C. After addition of 150 µL MTT solvent, plates were wrapped in foil and gently agitated for 15 min to dissolve formazan crystals. Absorbance was measured at 590 nm using a microplate reader (SPECTROstar Nano, CAT#0601-101; BMG LABTECH, Ortenberg, Germany).

### 4.7. Confocal Microscopy

Cell morphology and attachment were evaluated using confocal laser scanning microscopy (Zeiss LSM 700 microscope Carl Zeiss, Oberkochen, Germany). Scaffolds were seeded with 100,000 MC3T3-E1 pre-osteoblasts and cultured for 48 h to allow cell spreading and attachment. Cells were fixed in 3.7% methanol-free paraformaldehyde for 15 min at room temperature, permeabilized with 0.1% Triton X-100 for 15 min, and stained with Texas Red™-X Phalloidin (Thermo Fisher Scientific, USA) for F-actin and DAPI (4′,6-diamidino-2-phenylindole) for nuclei visualization. Samples were washed with PBS and mounted in antifade medium prior to imaging. Representative fluorescence images were acquired at 20× and 40× magnification to visualize cytoskeletal organization and overall cell morphology on the scaffold surfaces.

## 5. Conclusions

By leveraging a simple and experimentally accessible TIPS fabrication route, this study demonstrates that PCL/BaTiO_3_ composites can be processed into mechanically functional, highly porous three-dimensional scaffolds and clarifies how polymer and filler content govern pore morphology, mechanical response, and cellular behavior. Uniform BaTiO_3_ dispersion enhanced surface definition and introduced potential piezoelectric functionality without compromising cytocompatibility. Among the tested compositions, the 9% PCL/BaTiO_3_ formulation exhibited the most balanced performance. Future work will focus on quantifying electromechanical coupling and osteogenic potential under physiologically relevant mechanical loading.

## Figures and Tables

**Table 1:** Experimental and analytical elastic moduli of PCL and PCL + BaTiO_3_ scaffolds.

Specimen Type		*E* [MPa]		*f* *
		Experimental	Analytical	
Bulk	PCL	1.36	-	0%
	BaTiO_3_	90,000	-	0%
PCL	8%	1.82	0.024	87%
	9%	3.060	0.024	86%
	10%	1.49	0.031	86%
PCL/BaTiO_3_	8%	1.73	0.027	86%
	9%	0.68	0.035	84%
	10%	0.59	0.035	84%

* Equivalent pore volume fraction considering the entire specimen volume.

**Table 2:** Quantitative assessment of BaTiO_3_ surface coverage across scaffold formulations.

BaTiO_3_ Surface Coverage [%]	Polymer/Filler Content		
	8%	9%	10%
Mean	14.54%	13.42%	13.23%
Stdev	0.76%	0.58%	0.43%

**Table 3:** Mean optical density (OD_590_) values obtained from MTT assay for PCL and PCL/BaTiO_3_ scaffolds.

OD_590_ Values	Control	PCL			PCL/BaTiO_3_		
		8%	9%	10%	8%	9%	10%
Mean	0.268	0.246	0.268	0.247	0.224	0.238	0.248
Stdev	0.024	0.028	0.0136	0.046	0.008	0.014	0.045

## Data Availability

All data supporting the findings of this study are included within the article. Additional raw datasets generated and analyzed during the current work are available from the corresponding author upon reasonable request.

## Supplementary Materials

The following supporting information can be downloaded at: https://www.mdpi.com/article/10.3390/ijms27010406/s1.

## Abbreviations

The following abbreviations are used in this manuscript:

PCL	Polycarpolactone
FDM	Fused Deposition Modeling
BaTiO_3_	Barium titanate
TIPS	Thermally induced phase separation
THF	Tetrahydrofuran
