# Supplementary material for: Structure–Function Interplay in Piezoelectric PCL/BaTiO3 Scaffolds Fabricated by Phase Separation: Correlation of Morphology, Mechanics, and Cytocompatibility
Source: Int J Mol Sci. 2025 Dec 30;27(1):406. doi: 10.3390/ijms27010406 (PMC12785586; doi:10.3390/ijms27010406)
Supplement: Supplementary file 1 [file ijms-27-00406-s001.zip › ijms-4028891-supplementary.pdf]

The SEM micrographs in Figure S1 show that scaffolds fabricated at 5%, 6%, and 7% PCL concentrations exhibit a consistently porous and interconnected architecture across all magnification levels. At the microscale, pore size and pore-wall morphology appear broadly comparable among the three formulations, with no pronounced concentration-dependent differences in pore distribution or local surface texture. These observations indicate that thermally induced phase separation yields homogeneous porosity across this lower concentration range, despite the differences in macroscopic handling behavior discussed in the main manuscript.

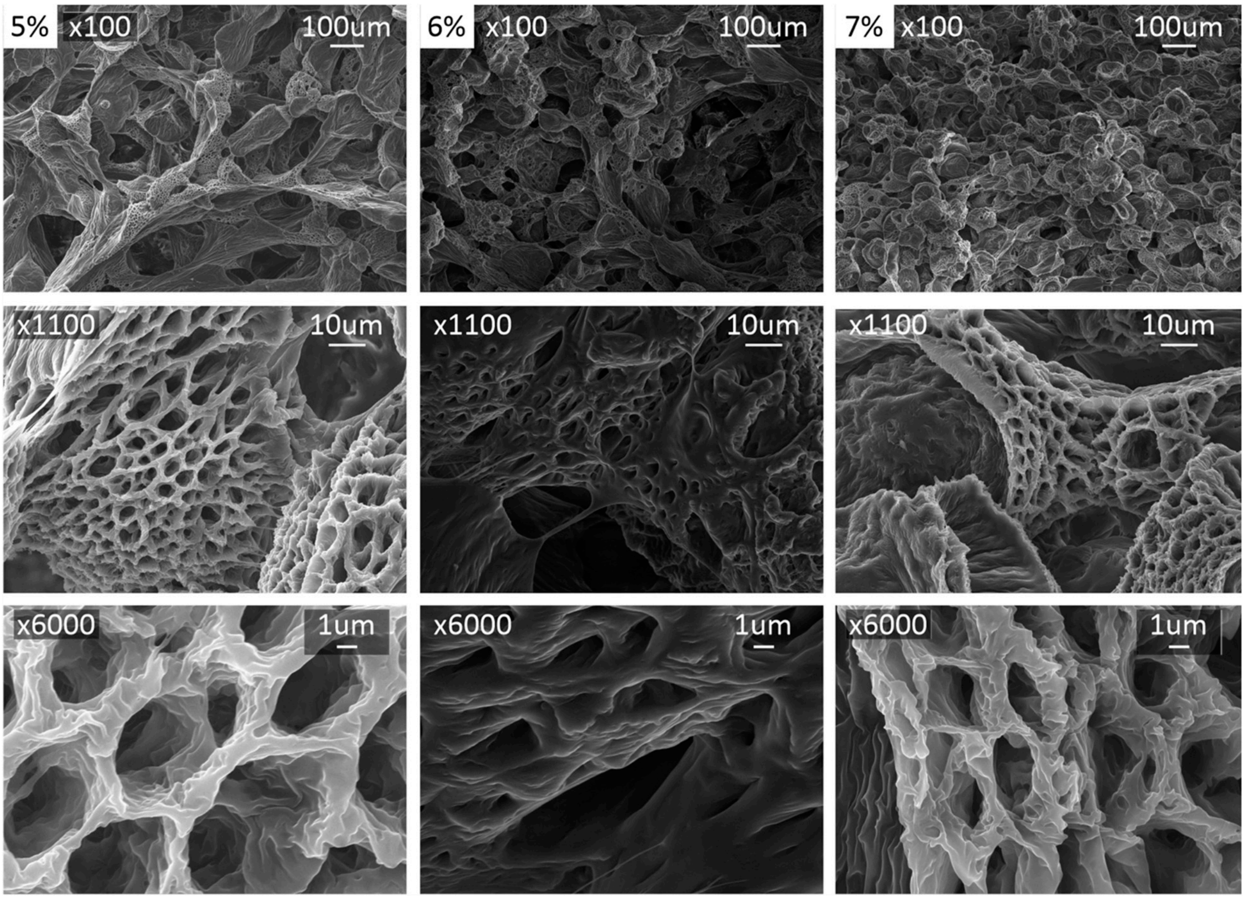

Figure S1. Representative SEM morphology of PCL scaffolds fabricated by TIPS at 5%, 6%, and 7% polymer-to-solvent concentrations. Consecutive magnifications are presented for each sample, with scale bars of 100 μm (100×), 10 μm (1100×), and 1 μm (6000×). Although porous architectures are observed at the microscale, these formulations exhibited compromised macroscopic structural integrity after demolding.
